# Periodontal Treatment for Preventing Adverse Pregnancy Outcomes: A Meta- and Trial Sequential Analysis

**DOI:** 10.1371/journal.pone.0129060

**Published:** 2015-06-02

**Authors:** Falk Schwendicke, Nadeem Karimbux, Veerasathpurush Allareddy, Christian Gluud

**Affiliations:** 1 Department of Operative and Preventive Dentistry, Charité —Universitätsmedizin Berlin, Berlin, Germany; 2 Tufts University School of Dental Medicine, Boston; 3 Department of Orthodontics, College of Dentistry—The University of Iowa, Iowa City; 4 Copenhagen Trial Unit, Centre for Clinical Intervention Research, Rigshospitalet, Copenhagen University Hospital, Copenhagen, Denmark; Columbia University, UNITED STATES

## Abstract

**Objectives:**

Periodontal treatment might reduce adverse pregnancy outcomes. The efficacy of periodontal treatment to prevent preterm birth, low birth weight, and perinatal mortality was evaluated using meta-analysis and trial sequential analysis.

**Methods:**

An existing systematic review was updated and meta-analyses performed. Risk of bias, heterogeneity, and publication bias were evaluated, and meta-regression performed. Subgroup analysis was used to compare different studies with low and high risk of bias and different populations, i.e., risk groups. Trial sequential analysis was used to assess risk of random errors.

**Results:**

Thirteen randomized clinical trials evaluating 6283 pregnant women were meta-analyzed. Four and nine trials had low and high risk of bias, respectively. Overall, periodontal treatment had no significant effect on preterm birth (odds ratio [95% confidence interval] 0.79 [0.57-1.10]) or low birth weight (0.69 [0.43-1.13]). Trial sequential analysis demonstrated that futility was not reached for any of the outcomes. For populations with moderate occurrence (<20%) of preterm birth or low birth weight, periodontal treatment was not efficacious for any of the outcomes, and trial sequential analyses indicated that further trials might be futile. For populations with high occurrence (≥20%) of preterm birth and low birth weight, periodontal treatment seemed to reduce the risk of preterm birth (0.42 [0.24-0.73]) and low birth weight (0.32 [0.15-0.67]), but trial sequential analyses showed that firm evidence was not reached. Periodontal treatment did not significantly affect perinatal mortality, and firm evidence was not reached. Risk of bias, but not publication bias or patients’ age modified the effect estimates.

**Conclusions:**

Providing periodontal treatment to pregnant women could potentially reduce the risks of perinatal outcomes, especially in mothers with high risks. Conclusive evidence could not be reached due to risks of bias, risks of random errors, and unclear effects of confounding. Further randomized clinical trials are required.

## Introduction

Bacterial infection and subsequent immunological reactions are hypothesized to cause adverse pregnancy outcomes like preterm birth, low birth weight, and perinatal mortality [1, 2]. Periodontal disease is discussed as one possible causal factor for adverse pregnancy outcomes, causing systemically increased levels of endotoxins, inflammatory cytokines, and oxidative stressors, which might negatively affect maternal and neonatal health [3, 4]. There is ambiguous evidence if mechanical subgingival debridement (‘periodontal treatment’), that is scaling and root planning, can reduce adverse pregnancy outcomes. A recently published systematic review and meta-analysis found significantly reduced risks of preterm birth (odds ratio (OR) [95% confidence interval] 0.66 [0.54–0.80]) and low birth weight (0.48 [0.30–0.78]) in populations with high occurrence of adverse pregnancy outcomes, whilst the estimates for those with moderate occurrence of adverse events and all patients were statistically non-significant [1]. Moreover, outcomes of periodontal treatment might be affected by patient- and study-related confounders, like age of the patient and risks of bias in the trials [1, 2]. Other reviews confirm the potential of periodontal treatment to reduce adverse pregnancy outcomes, but also raise doubts as to the generalizability of these findings and the level of reached evidence [1–4].

The evidence strength stemming from such traditional frequentistic meta-analyses might be additionally reduced as they do not fully account for random errors, that is spuriously significant results (type I errors) or spuriously insignificant results (type II errors), which may occur due to sparse data and repeated significance testing [5, 6]. Frequentistic meta-analyses ought to be based on an a priori calculated required information size (equivalent to the sample size in a randomized clinical trial), and meta-analyses conducted before the required information size is reached need to be assessed with more stringent statistical thresholds than usual in order to control spuriously significant results [5, 6]. Additionally, non-significance is often regarded as a matter of lack of effect, whilst truly, it may just be a type II error due to lack of statistical power before reaching the required information size [5, 6]. Trial sequential analysis accounts for such risks by calculating the required information size (RIS) and using trial sequential monitoring boundaries, which adjust significance levels depending on the data availability when testing for superiority [5, 6].

Since it remains unclear if periodontal treatment during pregnancy is efficacious to prevent preterm birth (PTB, <37th week), low birth weight (LBW, <2500 g), and spontaneous abortions or stillbirths (perinatal mortality, PNM), we conducted an updated meta-analysis, including a reassessment of bias risks and meta-regression to assess possible effects of confounders, and trial sequential analysis to assess the risks of random errors.

## Methods

### Randomized clinical trials and risk of bias

Our analyses were based on the review by Kim et al. including 11 randomized clinical trials [1]. Since the publication of this study, two more randomized clinical trials evaluating intra-pregnancy periodontal treatment for preventing adverse pregnancy outcomes have been published [7, 8]. Periodontal treatment was defined as subgingival scaling and root-planing with or without adjunctive use of antibiotics. The control group should have received all pre- and post-operative care (i.e., supragingival scaling, oral hygiene education, antibiotics) similarly to the experimental test group, only PT was not to be provided. PTB and LBW were assessed as events per total life births, where possible. We performed risk of bias assessment as outlined by the Cochrane Collaboration, trial with unclear or high risk of bias being assessed as trials with high risk of bias [9]. Whilst blinding of participants or personnel was assessed, this domain was not used for judging a trial’s overall risk of bias, since neither operators nor patients could have been ethically and effectively blinded. Risk of bias assessment was conducted by two authors independently, and a third author was contacted to reach consensus in case of disagreements.

### Meta-analysis and meta-regression

Conventional meta-analysis was performed with random-effects models using the DerSimonian-Laird estimator of variance [9], with odds ratio and 95% confidence intervals (95% CI) being calculated. Heterogeneity was assessed using both Cochrane’s Q and I^2^-statistics [10]. Publication bias was assessed graphically and statistically via funnel plot analysis and one-sided Egger’s regression test [11]. If publication bias was indicated, trim-and-fill was used for adjusting odds ratios [12].

Subgroups of trials with low and high risk of bias and trial with moderate (<20% for PTB, <20% LBW, <1% for PNM) or high (≥20% for PTB, ≥20% for LBW, ≥1% for PNM) control group event proportions were analyzed separately. Thus, the latter was used to define risk groups. It should be noted that such categorization is not biologically grounded, but has been used before to assess differential treatment effects of periodontal treatment on adverse pregnancy outcomes in different populations [1]. The used cut-offs had been chosen to allow balanced stratification of trials into subgroups for each outcome analysis. Such categorization does not build on any specifically outlined risk, as the reporting of potentially underlying risk factors is often incomplete, but uses the result of these unspecified risk factors, which were assumed to have been allocated to treatment and control group in a balanced manner due to randomization. Furthermore, subgroup-analyses were conducted for trials which did or did not perform any supragingival debridement in the control group, since such scaling might decrease any differences between the intervention groups [13]. Meta-regression-analysis was performed to assess the impact of participants’ average age on the effect estimates using the unrestricted maximum-likelihood method [14].

### Trial sequential analysis

The conventional meta-analysis uses Z-values to compare two interventions, with Z = 0 indicating no difference between groups [15]. If Z exceeds ±1.96, a difference is traditionally assumed to be statistically significant (p≤0.05, two-sided test). As for repeated updates of meta-analyses, a new Z-value is calculated for each update. In trial sequential analysis, this series of Z-values are plotted against the accumulated number of patients, outcomes, or information [5, 16]. This cumulative Z-curve is then assessed regarding its relation to the conventional significance boundaries (Z = ±1.96), the required information size, and the trial sequential monitoring boundaries (TSMB).

In trial sequential analysis, risk of type I error was set at α = 0.05. Risk of type II error was set at β = 0.20 equivalent to a power of 0.80. Relative risk reduction was defined a priori as a worthwhile interventional effect of 20% [16]. The resulting required information size was further heterogeneity-adjusted (HRIS) using the observed diversity [17].

The Lan-DeMets version [18] of the O’Brien–Fleming function [19] was used for calculating TSMB. Results crossing the conventional boundary of significance (Z = ±1.96) but not the superiority or inferiority TSMB were defined as spuriously significant. Firm evidence of superiority or inferiority was assumed to be reached when the Z-curve crossed the required RIS and the conventional boundaries hereafter or crossed the superiority or inferiority TSMBs before the required information size was reached. Firm evidence of futility was confirmed by the Z-curve crossing the futility TSBM. For meta-analysis or meta-regression-analyses, Comprehensive Meta Analysis (2.2.064, Biostat, Englewood, NJ, USA) was used. For trial sequential analysis, TSA 0.9 (Copenhagen Trial Unit, Copenhagen, Denmark) was used [6, 20].

## Results

A total of 13 randomized clinical trials evaluating 6283 pregnant women were included. All trials provided data regarding PTB and PNM, whilst only 10 provided data regarding LBW. Attrition was 0% to 11%, with 6037 observations remaining for follow-up. Four trials were judged as having low risk of bias, the others as having high risk of bias (Table 1).

**Table 1:** Risk of bias of the included trials.

Study	Sequence generation	Allocation concealment	Blinding of participants and personnel*	Blinding of outcome assessment	Incomplete outcome data assessed	Selective reporting	Free of other bias	Overall risk
Lopez 2002 [39]	Low risk of bias. (Randomization by toss of coin.)	Unclear risk of bias.(No mention of concealment.)	High risk of bias.(No blinding strategy.)	Low risk of bias.(Obstetrician masked.)	Low risk of bias.	Low risk of bias.	Low risk of bias.	High risk of bias.
Jeffcoat 2003 [40]	Low risk of bias. (Randomization accomplished by code.)	Low risk of bias.(Allocation concealment by use of double packet with coding information.)	Low risk of bias. (Mention about blinding in study design.????? Can this become more clear?????)	Low risk of bias. (Mention about blinding in study design.)	Low risk of bias.	Low risk of bias.	Low risk of bias.	Low risk of bias.
Sadamansouri 2006 [41]	Unclear risk of bias.(No mention about how randomization was accomplished.)	Unclear risk of bias.(No information on allocation concealment.)	High risk of bias.(No mention about blinding.)	High risk of bias.(No mention about blinding	Unclear risk of bias.(Effects of covariates and confounders on outcomes are not examined.)	Low risk of bias.	Unclear risk of bias.(Imbalance in baseline characteristics.)	High risk of bias.
Offenbacher 2006 [42]	Unclear risk of bias. (No mention about how randomization was accomplished.)	Unclear risk of bias (No information on allocation concealment.)	Low risk of bias.(Mention about examiners being blinded.)	Low risk of bias.(Mention about examiners being blinded.)	Low risk of bias	Low risk of bias.	Unclear risk of bias. (Periodontal status imbalanced at baseline.)	High risk of bias.
Michalowicz 2006 [43]	Low risk of bias. (Block randomization.)	Low risk of bias. (Mention about telephone calls and central randomization.)	Low risk of bias.(Mention about blinding in study design.)	Low risk of bias.(Mention about blinding in study design.)	Low risk of bias.	Low risk of bias.	Low risk of bias.	Low risk of bias.
Tarannum and Faiduzzin 2007 [44]	Low risk of bias. (Randomization by flip of coin.)	Unclear risk of bias. (No mention about allocation concealment.)	High risk of bias.(No information on blinding strategy is provided.)	High risk of bias.(No mention about blinding.)	Low risk of bias.	Low risk of bias.	Low risk of bias.	High risk of bias.
Radnai 2009 [45]	Low risk of bias. (Block randomization by of random sequence of numbers.)	Unclear risk of bias. (No mention about allocation concealment.)	Low risk of bias.(Mention about blinding in study design.)	Low risk of bias.(Mention about blinding in study design.)	Low risk of bias.	Low risk of bias.	Low risk of bias.	High risk of bias.
Newnham 2009 [46]	Low risk of bias. (Randomization accomplished by computer generated software.)	Unclear risk of bias.(No mention about allocation concealment.)	High risk of bias.(No information on blinding strategy is provided.)	Low risk of bias.(Assessors unaware of treatment.)	Low risk of bias	Low risk of bias.	Low risk of bias.	High risk of bias.
Offenbacher 2009 [47]	Low risk of bias. (Permutated block randomization.)	Unclear risk of bias (no mention about allocation concealment)	High risk of bias.(No information on blinding strategy is provided.)	Low risk of bias.(Dental examiners were blinded.)	Unclear risk of bias. (Unclear how missing data was treated.)	Low risk of bias.	Unclear risk of bias. (Baseline characteristics similar excepting for number of nulliparous pregnancies and self-reported alcohol use.)	High risk of bias.
Oliveira 2011 [48]	Unclear risk of bias. (Not clear how randomization was accomplished.)	Unclear risk of bias.(No mention about allocation concealment.)	High risk of bias. (No information on blinding strategy is provided.)	Low risk of bias.(Mention about blinding in study design.)	Low risk of bias.	Low risk of bias.	Unclear risk of bias. (Differences in periodontal status at baseline.)	High risk of bias.
Macones 2011 [49]	Low risk of bias. (Permutated block randomization.)	Unclear risk of bias. (No mention about allocation concealment).	High risk of bias.(No information on blinding strategy is provided.)	Low risk of bias.(Outcome assessors blinded.)	Low risk of bias.	Low risk of bias.	Low risk of bias.	High risk of bias.
Pirie 2013 [8]	Low risk of bias. (Randomization accomplished by computer generated random number.)	Low risk of bias.(Allocation concealment accomplished by use of opaque sealed envelope.)	Low risk of bias. (Staff members were masked for treatment.)	Low risk of bias. (Staff members were masked for treatment.)	Low risk of bias.	Low risk of bias.	Low risk of bias.	Low risk of bias.
Weidlich 2013 [7]	Low risk of bias. (Randomization accomplished by computer generated random number-block stratification.)	Low risk of bias. (Allocation concealment accomplished by use of sealed envelope.)	High risk of bias.(No information on blinding strategy is provided.)	Low risk of bias.(Independent examiners.)	Low risk of bias.	Low risk of bias.	Low risk of bias.	Low risk of bias.

* Risk of bias stemming from not blinding participants or personnel was not used to decide a trial’s overall risk of bias.

**Fig 1:**
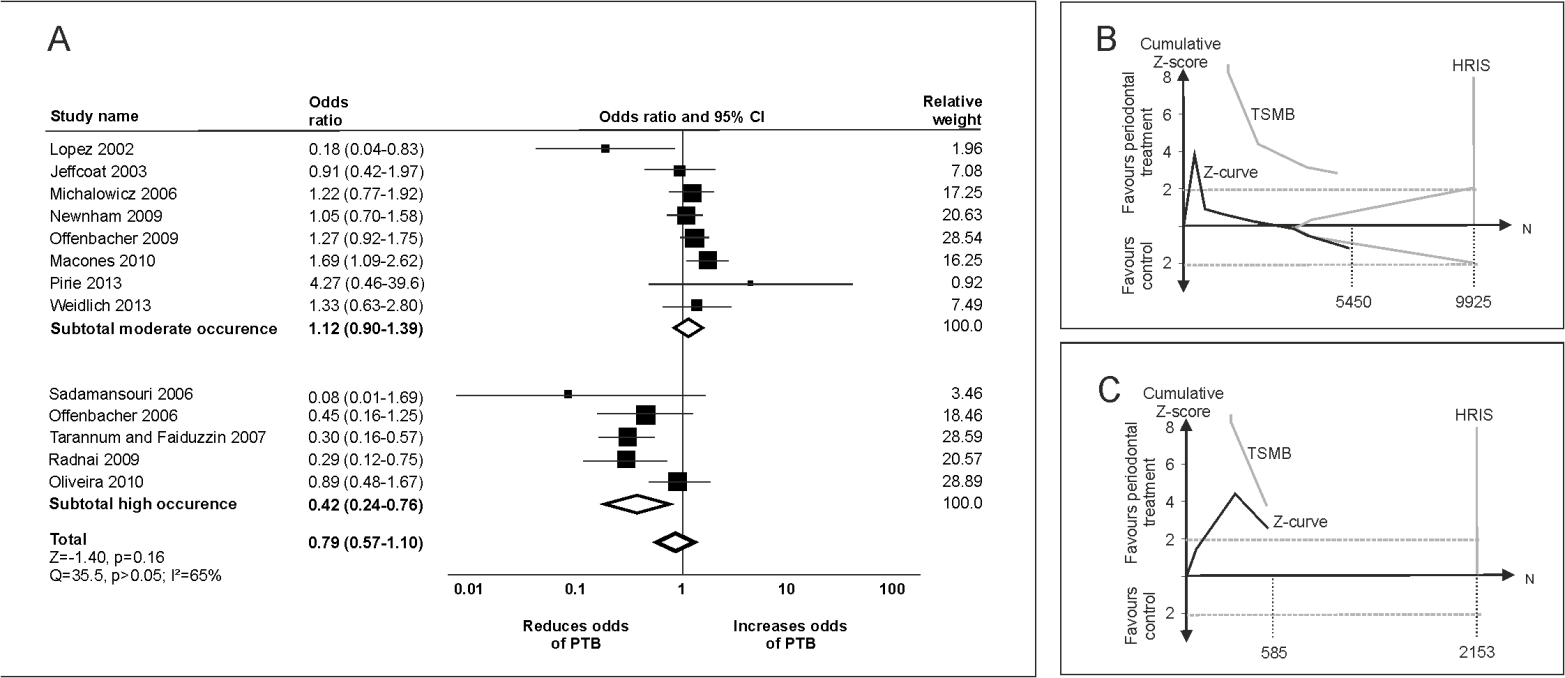
Periodontal treatment and preterm birth (PTB). (A) Conventional meta-analyses were performed to analyze the effect of periodontal treatment in control populations with moderate (<20%) and high occurrence (≥20%) of PTB as well as in the total population. Heterogeneity was assessed using χ^2^-test and I^2^-statistics. (B) Trial sequential analysis of trials in population with moderate occurrence of PTB. The cumulative Z-score (black), i.e., the accumulated level of significance, was plotted against the number of participants accrued so far, which was compared with the heterogeneity-adjusted required information size (HRIS). Based on HRIS, the trial sequential monitoring boundary (TSMB) for benefit was plotted (grey oblique). The Z-curve nearly crosses the futility boundary, and HRIS is not reached. (C) Trial sequential analysis of trials in population with high occurrence of PTB. The Z-curve does not reach the HRIS, and does not cross the TSMB.

For PTB, conventional meta-analysis did not find a significant effect of periodontal treatment (OR [95% confidence interval] 0.79 [0.57–1.10]; Fig 1A). Both funnel plot analysis and Egger’s-test (p<0.05) indicated publication bias (adjusted OR 0.85 [0.60–1.21]). Trials with low risk of bias did not show a significant effect of periodontal treatment (OR 0.96 [0.54–1.69]) a finding replicated in trials with high risk of bias (OR 0.70 [0.46–1.08]). Patients’ age did not significantly affect the effect estimates (p>0.05). From the HRIS of 19,656 participants, only 31% (6035) was accrued, with no firm evidence being available. If only populations with moderate occurrence of PTB (<20%) were evaluated, periodontal treatment was not efficacious (OR 1.12 [0.90–1.39]), and based on trial sequential analysis, future trials might not be able to prove superiority of periodontal treatment in comparison with placebo for reducing PTB (Fig 1B). In populations with high occurrence of PTB (≥20%), periodontal treatment seemed to significantly decrease the risk of PTB (OR 0.42 [0.57–0.73]), but firm evidence of superiority of periodontal treatment was not reached (Fig 1C).

**Fig 2:**
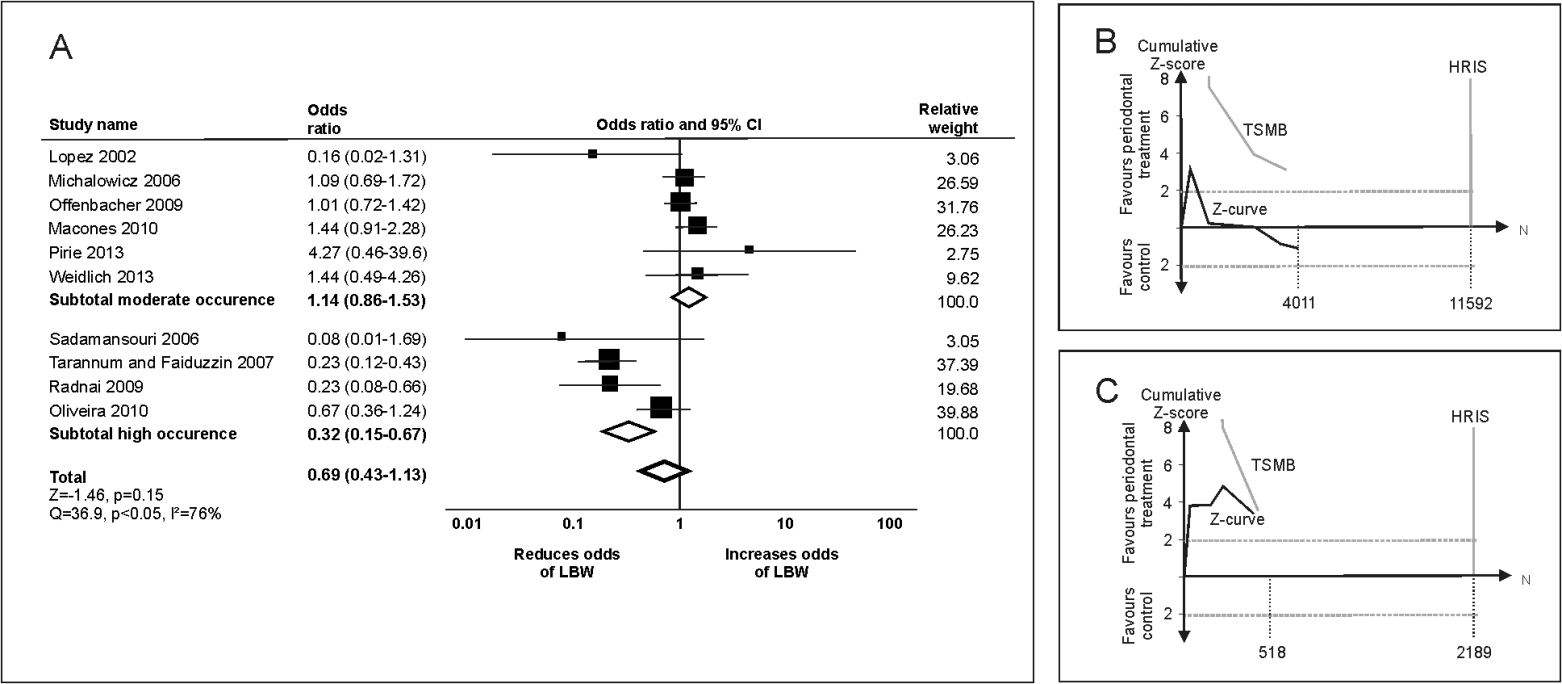
Periodontal treatment and low birth weight (LBW). (A) Conventional meta-analyses were conducted to analyze the effect of periodontal treatment for populations with moderate (<20%) and high occurrence (≥20%) of LBW as well as in the total population. (B) Trial sequential analysis of trials in population with moderate occurrence of LBW. The Z-curve only initially crosses the conventional boundary, with trial sequential monitoring boundary (TSMB) for benefit and HRIS not being in reach. (C) Trial sequential analysis of trials in population with high occurrence of LBW. The Z-curve does not reach the HRIS, and does not cross the TSMB.

For LBW, periodontal treatment had no significant effect (OR 0.69 [0.43–1.13]; Fig 2A). Funnel plot analysis, but not Egger-test indicated risk of publication bias (p>0.05). The adjusted estimate remained non-significant (adjusted OR 0.73 [0.45–1.19]). Trial with low risk of bias showed no significant effect of periodontal treatment on LBW (OR 0.92 [0.37–2.31]) in contrast to trials with high risk of bias (OR 0.58 [0.30–1.13]). This difference was significant (p<0.05). Patients’ age did not significantly modify the estimate (p>0.05). From the HRIS of 22,476 participants, only 20% (4529) was accrued, with no firm evidence being available. In populations with moderate occurrence of LBW, periodontal treatment was not found to significantly affect risk of LBW (OR 1.14 [0.86–1.53]). Trial sequential analysis found no firm evidence to be currently available supporting or refuting periodontal treatment in such groups (Fig 2B). For populations with high occurrence of LBW, periodontal treatment seemed to significantly decrease LBW (OR 0.32 [0.15–0.67], but firm evidence was not reached (Fig 2C).

**Fig 3:**
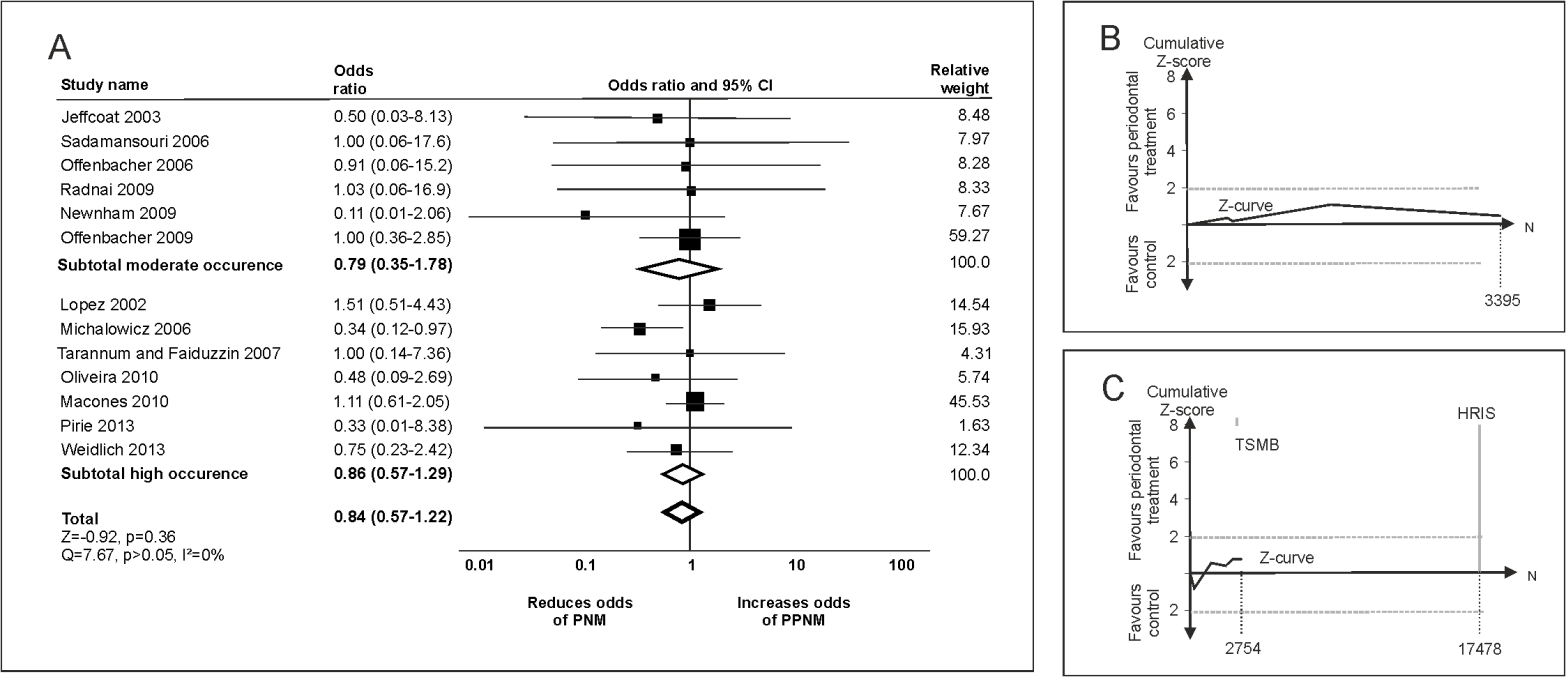
Periodontal treatment and perinatal mortality (PNM). (A) Conventional meta-analyses were conducted to analyze the effect of periodontal treatment for populations with moderate (<1%) and high occurrence (≥1%) of PNM as well as in the total population. (B) and (C) Trial sequential analyses of trials in populations with moderate and high occurrence of PNM, respectively. The conventional boundary, the trial sequential monitoring boundary (TSMB) for benefit, the TSMB for futility, and the HRIS are not reached.

Periodontal treatment was not found to significantly reduce the risk of PNM (OR 0.84 [0.57–1.22], Fig 3A). Funnel plot, but not statistical analysis indicated publication bias (p>0.05). The adjusted effect estimate remained non-significant (adjusted OR 0.88 [0.61–1.26]). Neither risk of bias nor patients’ age significantly modified the effect estimate. From the HRIS of 33,003 participants, only 19% (6149) was accrued, with no firm evidence being available. Neither in groups with low (<1%) nor high (≥1%) occurrence of PNM did periodontal treatment significantly change the risk (OR 0.79 [0.35–1.78] and OR 0.86 [0.57–1.29], respectively). In trial sequential analysis, the Z-curves never crossed the traditional significance boundaries, and no firm evidence was reached neither for populations with moderate (Fig 3B) nor high occurrence (Fig 3C).

Regardless of the outcome, excluding trials which had performed supragingival scaling in the control group did not significantly change the intervention effect estimates (data not shown).

## Discussion

Traditional meta-analysis might be prone to random errors, especially when evaluating results of only few early trials with limited quality and small number of patients [2]. In addition, repeated significance testing when updating meta-analyses might generate erroneous results [5]. Using both conventional meta-analysis and trial sequential analysis, we show that the general provision of periodontal treatment does not seem to prevent adverse pregnancy outcomes. Based on our results, it seems unlikely that firm evidence for such an efficacy will ever be reached, whilst possible harmful effects cannot be ruled out. Only for high-risk populations, periodontal treatment appeared potentially efficacious to prevent PTB and LBW, supporting results from previous analyses [1, 2], whilst trial sequential analysis indicated that firm evidence for this efficacy has not been established so far. For PNM, periodontal treatment was generally not found efficacious, but trial sequential analysis demonstrated that we have very sparse data on this outcome.

For LBW and PNM, risk of bias of included trials significantly modified the effect estimates. It can be argued that none of the trials was of true high risk of bias, as the domain mostly leading to downgrading of trials to ‘high’ risk of bias was lack of blinding of examiners, i.e., there was a risk of detection bias. The ascertainment of outcomes like PTB or PNM is most likely not prone for such bias. However, most trials lacked sufficient allocation concealment as well, and all included trials might be affected by risk of academic or professional bias [9, 21], which was not judged within our study. Such bias is overestimation of benefits and underestimation of harms comparable to industry bias [21], with the difference that explicit and direct financial interests are not present, whilst both indirect financial or non-financial conflicts of interest (competition for public extra-mural support, academic competition, intellectual passion) might be present, which are far more difficult to assess [22–24]. Considering these limitations and our results, the summarized evidence is insufficient to support or refute periodontal treatment for reducing adverse pregnancy outcomes. Our findings add weight to previous studies [25–29], which doubted the association between periodontal treatment and PTB or LBW found by observational studies [30, 31]. Possible reasons for the discrepancy between results from observational studies and randomized clinical trials have been discussed elsewhere [25, 31].

The present study has several limitations. First, we performed an update of an existing review, with the inherent risk of having missed studies, i.e., introducing selection bias. Our study should therefore not be regarded as a systematic review, but as an updated meta-analysis, with the strength of using a rigorous method for assessing the evidence reached so far, but the weakness of lacking a systematic screening of studies. We have checked for the potential bias of missing or non-published studies and did not find this to affect our estimates. Second, the used methods for evaluating the effects of such publication bias (trim-and-fill) should be regarded with caution as well. Third, we did not exhaustively analyze all possible confounders like common risk factors or baseline periodontal condition, since extensively performing further subgroup- and sensitivity-analyses would re-introduce the risk of type I and type II errors, which we attempted to tackle using trial sequential analysis. One confounder with potential effects on the efficacy of periodontal treatment for reducing adverse pregnancy outcomes is the provided care itself. The efficacy of this care has not been demonstrated throughout all included trials (as not all report on a sufficient set of valid outcomes of periodontal treatment), and different procedures (non-surgical and surgical, with and without concomitant antibiotic treatment) and supportive treatments have been provided [32]. Thus, besides having potentially different effects in different risk groups, the differential provision of periodontal treatment might also impact on its efficacy for reducing adverse pregnancy outcomes. In future trials, the resolve of the disease and the associated signs of inflammation should be reported in sufficient detail to assess the clinical impact of periodontal treatment. This also applies to a third confounder. The timing of periodontal treatment might determine both its efficacy for reducing signs and symptoms of periodontal disease and the associated inflammatory response, thus also impacting on the potential efficacy for reducing adverse pregnancy outcomes [32].

In conclusion, the main indication for providing periodontal treatment during pregnancy should be periodontal disease itself [33, 34]. For further recommendations, possible harmful effects should be first ruled out, and benefits of periodontal treatment should be rigorously demonstrated. To do so, future randomized clinical trials should aim at assessing relevant confounders, provide efficacious periodontal treatment, and control the effects of this care on the signs and symptoms of the disease. Multi-center international trials with blinded and independent outcome examination might be suited to reach both the required quality and information size. Such trials should primarily be conducted in participant groups with high risks, and methods for reliable identification of such populations should be identified [35–38].
